# Promoting Success and Persistence in Pandemic Times: An Experience With First-Year Students

**DOI:** 10.3389/fpsyg.2022.815584

**Published:** 2022-03-03

**Authors:** Joana R. Casanova, Alexandra Gomes, Maria Alfredo Moreira, Leandro S. Almeida

**Affiliations:** ^1^Research Centre on Education (CIEd), Institute of Education, University of Minho, Braga, Portugal; ^2^Research Centre on Child Studies (CIEC), Institute of Education, University of Minho, Braga, Portugal

**Keywords:** higher education, adaptation to university, first-year students, COVID-19 pandemic, dropout

## Abstract

The transition and adaptation of students to higher education (HE) involve a wide range of challenges that justify some institutional practices promoting skills that enable students to increase their autonomy and to face the difficulties experienced. The requirements for this adaptation were particularly aggravated by the containment and sanitary conditions associated with coronavirus disease 2019 (COVID-19). With the aim of promoting academic success and preventing dropout in the first year, a support program was implemented for students enrolled in two courses in the area of education at a public university in northern Portugal during the first semester of 2020/2021. Three sessions of 50/60 min were implemented, namely, the first session focused on the verbalization of the demands, challenges, and difficulties of the transition, and the second and third sessions focused on the difficulties of academic adaptation and academic performance. Data from a dropout risk screening instrument and from the activities performed during sessions were analyzed. The main results point to student satisfaction with the content and the activities of the sessions and their usefulness. Students report not only high satisfaction levels with HE attendance, but also some emotional exhaustion due to academic activities. The continuity of the program is recommended with some improvements in its planning to ensure a more definitive version of the program in the next two years.

## Introduction

The European Education Area that was implemented some years ago seeks to foster cooperation between European Union Member States to enhance the quality and inclusiveness of national education and training systems (Farnell et al., 2021). The educational perspective based on the European Credit Transfer System has led to changes in the teaching-learning processes and requires the development of self-regulation skills, transversal, and lifelong education competencies by the students (Esteban et al., 2017; Panadero, 2017; van Rooij et al., 2018). To address these goals, the member states are improving their educational policies to increase the number of students in the tertiary education system and to reduce the number of students who drop out before graduation (OECD, 2018).

In Portugal, the expansion of higher education (HE) in recent decades has reflected the increase in the attendance of ethnic and socio-cultural groups that were previously excluded. However, this democratization is only reflected in a wider access but not in students’ retention and success rates. Students who are less mature and less prepared academically present several difficulties when facing the demands or challenges of HE. Students who do not enter HE immediately after secondary education completion or who enter HE after several years in the labor market, students from disadvantaged socioeconomic groups or first-generation students in HE, migrants or students belonging to minority ethnic groups, among others, are the least equipped to successfully face the challenges of HE (Adabaş and Kaygin, 2016; Tight, 2019; Casanova et al., 2021c). In Portugal, students from low socioeconomic groups are more represented in polytechnic schools or in social and human sciences programs at university, seldom in their first vocational option (nearly 40%), as the entry system based on grades strongly regulates access to HE (Fonseca et al., 2014; Ferrão and Almeida, 2018; OECD, 2019). This socioeconomic differentiation in access persists in their academic trajectories in undergraduate programs, as students from low socioeconomic groups present higher academic failure and dropout rates.

In this article, the objectives, activities, and results of a pilot program are presented to advance a definitive version in the future. This effort is based on the awareness that supporting first-year students in their academic adaptation and success should happen through institutionally supported programs, avoiding short-time efforts, and preventing the end of assistance due to the departure of the responsible person, end of funding, changes in administration, or other motives (Tinto, 2012). A successful intervention program takes time and should respect the sociocultural characteristics of the institution and community in which it occurs, always with the main goal of addressing their real needs.

The HE withdrawal affects the individual, the educational institution, and the society at large (Duque, 2014; Casanova et al., 2018; Ferrão and Almeida, 2021). Vincent Tinto (1975) presented the first model of student attrition that is widely used in explaining the dropout phenomena, considering social and academic integration at university. His holistic approach points to teachers, academic staff, and peer relationships as relevant variables in the process of dropout decision-making. Research in this area evidences that student dropout can be related to personal characteristics (e.g., abilities, competencies, and motivations), family characteristics (e.g., first-generation students, sociocultural, and socioeconomic situation), academic background (e.g., previous achievement and vocational choices), and contextual characteristics (e.g., curriculum organization, size, and quality of institutions), among other (Häfner et al., 2015; Lindblom-Ylänne et al., 2017; Casanova et al., 2021b; Wangrow et al., 2022).

Often, students’ levels of autonomy are not sufficient to face challenges and demands that characterize the HE context (Pascarella and Terenzini, 2005; Kuh et al., 2008). These levels of autonomy can be developed with some support, to address the responsibilities and self-determination of students in their academic activities. Teachers can promote students’ self-determination, thus favoring their active learning behaviors (Ryan and Deci, 2000). This influence is evident through teachers’ support in promoting their students’ autonomy and in creating positive learning environments, e.g., helping students to overcome situations of anxiety or depression (Skinner et al., 2009; Lei et al., 2018). Teachers’ support impacts students’ academic emotions, increasing the positive emotions (e.g., enjoyment and hope) and decreasing the negative emotions (e.g., anxiety and anger), which may be decisive to ensure students’ permanence and success during their first weeks in HE. In preventing academic failure and dropout, it is important to consider students’ feelings of belonging to the institution and course, as well as good teacher-student interactions and positive peer relationships. Friendly relationships, including social, emotional, and appraisal support from teachers, allow students to increase academic engagement and to develop positive emotions, such as satisfaction, psychological wellbeing, and enjoyment with the academic experience (Respondek et al., 2017; Lei et al., 2018; Casanova et al., 2021a). These aspects should be considered in programs to promote students’ competencies to ensure a positive transition and adaptation to HE.

Academic achievement stands out as a key factor in dropout prediction, namely, in the first-year students (Pascarella and Terenzini, 2005; Ayala and Manzano, 2018; Díaz-Mujica et al., 2019; Casanova et al., 2021a; Ferrão and Almeida, 2021). Thus, learning strategies and personal characteristics, such as expectations, self-efficacy, conscientiousness, or autonomous motivation, are included in the programs to promote students’ academic success and retention (Kuh et al., 2008; Diniz et al., 2018; Girelli et al., 2018; Sobowale et al., 2018; Stajkovic et al., 2018).

The institutional implementation of a program that promotes success, engagement, and retention is quite challenging because of the diversity of students and their needs. The development of this type of program should be based on a deep knowledge of the students’ characteristics and experiences (e.g., previous academic trajectories, motives to entering HE, sociocultural characteristics, motives for considering dropping out, and academic and social difficulties) and the resources available and the value assigned by students. Despite the relevance of initiatives, such as summer programs, workshops, and specialized services that institutions can offer, a broader and more democratic way to promote students’ participation, success, and persistence is through initiatives in the classroom (Tinto, 2012). In this way, promoting adaptation and retention will reach those who do not have time beyond classes, nor the autonomy or agency to engage in other activities besides the mandatory ones (Tinto, 2012; Wangrow et al., 2022).

The recent pandemic situation resulted in a heightened awareness of the impact of dropout in students’ and their family’s living conditions, and its consequences for societal development. Coronavirus disease 2019 (COVID-19) had a relevant impact on teaching and learning activities and, therefore, on students’ academic experiences and achievement (Schleicher, 2020; Sáiz-Manzanares et al., 2022). Many students perceived a decrease in the quality of their learning, namely, in what comes to practical classes, lab, and practicum activities, regarded as critical for their professionalization, while others anticipated failing in the academic year (Hasan and Bao, 2020; Farnell et al., 2021). Students from less privileged families present more difficulties to face the severe limitations of lockdown, and fewer opportunities for remaining engaged and for investing in learning activities. Quality of internet connection and technological equipment or students’ digital competencies differentiated learning experiences during the pandemic (Schleicher, 2020; Sáiz-Manzanares et al., 2022). In Portugal, the conditions to fully participate in remote teaching sessions affected mainly students from rural areas and from socially disadvantaged groups.

Coronavirus disease 2019 also impacts students’ socioemotional adaptation and mental health, especially due to the uncertainty about the disease, about the national and local resources, and about personal and one’s family’s physical and mental health (Wang and Zhao, 2020; Farnell et al., 2021). This uncertainty fuels feelings of fear and uncontrollability of life, as the brutality of the pandemic has impacted all countries, including highly developed ones, directly affecting people who had never thought that they would be facing such hardships. It is important to recognize that a review of international research, prior to COVID-19, had already alerted to the high prevalence of symptoms of psychological distress in university students, with symptoms of anxiety in more than 40% and of depression in more than 25% (Cvetkovski et al., 2018). COVID-19 led to higher rates of consumption of psychoactive substances or psychopathologies, associated with experiences of loneliness, fear, and depression (Wang and Zhao, 2020; Charles et al., 2021; Schiff et al., 2021). The illness and death of family members, the closing of gyms and sports spaces and the consequent reduction in physical activity, less frequent and only remote relationships with friends, or the poor quality of equipment used to attend classes reduced the wellbeing of students (Maugeri et al., 2020).

The COVID-19 crisis has also led to an increase in academic difficulties. The universities have made a serious effort to implement alternatives to the absence of face-to-face teaching, to promote remote teaching and learning activities, as well as to ensure psychosocial support services. Considering the first-year students in mind, different responses are organized by institutions to facilitate their transition and adaptation. In general, institutions organize a reception day or week, providing information about institutional services and program organization and teaching activities, usually resorting to groups of students, teaching staff, and the program director. In a more targeted way, more specific measures are implemented, according to the type of incoming students and their educational or psychosocial needs. Study competencies and organization of curricular activities, programs for the development of interpersonal competencies, short or semester courses for leveling knowledge assumed as a requisite for key curricular units, activities for the development of the student’s daily functional autonomy (e.g., funds and time management), or career counseling and career development sessions, among other activities, illustrate the diversity of preventive or promotional programs. Economic difficulties, loneliness or lack of social support, low self-efficacy in learning strategies, anxiety and depression, frustration of initial expectations, feelings of maladjustment, and psychological discomfort are often found in the first weeks at university (Araújo et al., 2016; Araújo, 2017; Díaz-Mujica et al., 2019; Sáiz-Manzanares et al., 2022). When experienced, such difficulties are either quickly and effectively overcome or tend to favor the behaviors of progressive disengagement from academic life, generating emotional exhaustion, failure in learning, and risk of dropping out.

This article describes the initial phase of a program developed to promote academic adaptation, learning achievement, and persistence in first-year students from Education degrees in a Portuguese public university during the pandemic crisis. Previous studies (ObservatoriUM, 2017, 2018) identified this group as displaying some adaptation risk because of their general profile, namely, low socioeconomic family status, family without a tradition of HE, leaving home to live near the university, not attending a degree of first vocational option, lower grade point average (GPA) in access, or nearly 20% of first-year dropouts. Considering these characteristics and the expected negative COVID-19 impact, a promotional program was developed to support first-year students from Education degrees in their academic adaptation and academic achievement.

## Materials and Methods

### Participants

This study considers first-year students in two undergraduate programs in the scientific area of education, namely, Basic Education and Education (daytime and after-work). The annual reports produced by ObservatoriUM – Observatory of the Academic Paths of Students at the University of Minho (2017, 2018) suggest several academic vulnerabilities of students from Education programs. They are mostly students who benefit from a scholarship (awarded according to household income), and their parents’ academic qualifications are low (parents with HE level never exceed 6% and mothers with HE do not usually exceed 20%). They are, therefore, mostly a group of first-generation students, with no family tradition of attending HE, which can foresee less preparation and resources to face the demands of this new education level. This sociocultural profile could account for the fact that, prior to the pandemic, nearly 20% of students in education courses drop out during their first year in HE.

One hundred and fifty students from the Basic Education and Education programs participated in the intervention sessions. There were two groups of students from Basic Education and three groups from Education (two groups of daytime students and one group of after-work students). Given the developmental and personal nature of the activities carried out in the sessions, we intentionally did not request microdata from students (e.g., age, gender, and socio-familiar descriptions) to ensure free and active participation in the activities.

### Intervention Sessions

The intervention program to promote academic adaptation, academic achievement, and persistence was implemented in collaboration with the Pedagogical Council, a board that includes both teaching staff and students representatives. The first author, a psychologist, and researcher in the area of HE dropout, held three intervention sessions for the five groups of students. Each session lasted approximately 1 h and took place in October and December 2020. Table 1 presents the sessions’ themes, aims, and information collected.

**TABLE 1:** Sessions, aims, and information collected.

Session	Aims	Information collected and analyzed
“Being a Higher Education student: the importance of a good start”	To facilitate students’ identification of challenges and opportunities for learning and wellbeing	Academic challenges Threats to wellbeing Opportunities to enhance learning
“Being a Higher Education student in times of pandemic: academic achievement and success”	To address academic satisfaction, involvement in learning and in the relationship with colleagues and teachers	Well-managed personal and academic situations and tasks Challenging situations and tasks Strategies to manage uncertainty
“Being a Higher Education student: how it is going”	To analyze academic experiences like classes and learning, first results on academic achievement, satisfaction or dropout intentions	Threats to academic performance and achievement and wellbeing Thoughts about leaving Strategies to manage uncertainty and promote persistence

The first (face-to-face) session, entitled “Being a Higher Education student: the importance of a good start,” introduced several activities to facilitate students’ identification of challenges and opportunities for learning and wellbeing in HE. The second session (online), entitled “Being a Higher Education student in times of pandemic: academic achievement and success,” addressed academic satisfaction, involvement in learning, and the relationship with colleagues and teachers during the first weeks in HE. The third session (also online), entitled “Being a Higher Education student: how it is going,” analyzed academic experiences, such as classes and learning, first academic achievement results, satisfaction, and dropout intentions after the first exams.

### Instruments and Digital Resources

Because of the increasing sanitary risk, there was only a face-to-face session. The remaining online sessions were held in *Wooclap*^1^, a tool with several features for interactive and collaborative learning and compatible with the majority of digital resources used in HE. Each session started by asking students about difficulties in academic adaptation, peer relationships and support, and thoughts of leaving. Students answered to a screening instrument for students at-risk of dropping out from HE (Casanova et al., 2021c). It was composed of 12 items that were distributed by three scales, namely, (i) Satisfaction with education (4 items, e.g., I am satisfied with the education I am receiving at this university), (ii) Academic exhaustion (4 items, e.g., I feel exhausted due to my course activities), and (iii) Dropout intention (4 items, e.g., I am thinking of leaving HE). The first two scales were applied in all three sessions, being the scale of dropout intention only applied in the third session after students had received the results of the mid-term exams. The items were answered on a 5-point Likert-type agreement scale. The scales show adequate reliability coefficients, namely, Satisfaction with education (α = 0.81), Academic exhaustion (α = 0.83), and Dropout intention (α = 0.76). Confirmatory factor analysis confirmed the differentiation of these three constructs, and structure measure invariance by gender was also ensured (Casanova et al., 2021c).

### Procedures

This study was conducted in accordance with the Ethical Standards for Research with Humans and is part of a larger research project concerning dropout prevention in first-year university students, previously approved by the University Ethics Committee (CEICSH035/2019).

In the first session, being in the classroom ensured that students would be available to carry out the activities, since it was a period already destined for face-to-face academic tasks. Students were informed of the project, the three sessions, and their objectives, and gave free and informed written consent to participate in the project. Students were assured of the confidentiality of the data that they were not obliged to participate or continue with the study, and could leave the study whenever they chose.

At the beginning of the sessions, students connected with the *Wooclap* tool to participate, and their input was visible to everyone in the class. Initially, students answered a set of screening risk items about their satisfaction with education and academic experiences, as well as possible academic exhaustion. This was the starting point to naming, normalizing, and validating students’ emotions and perceptions, both positive and negative ones. In a collaborative way, the researchers and students co-constructed these moments with some input about what the scientific literature and the institutional data from previous years state about this period at university. Practical activities were implemented, creating space for peer reflection and sharing in a relaxed and immediate way of communicating. The aim of this study was to promote self-awareness, autonomy competencies, and self-efficacy, as well as the exploration of alternative solutions for personal difficulties experienced. The acquisition of several problem-solving competencies was also developed. The sessions ended with an evaluation moment, when participants would point their satisfaction level with the contents, activities, and processes.

### Data Analysis

Data were extracted from a Wooclap datasheet, exported, and analyzed using the IBM-SPSS Statistics version 28 for descriptive statistical analysis. Words mentioned by the students for characterizing the meaning of attending HE (co-construction of a word cloud) were analyzed in terms of frequencies, but also in a more qualitative approach, namely, by themes and meaning, both positive and negative meanings, as well as feelings.

## Results

In the first session (“Being a Higher Education student: the importance of a good start”), students described their experiences in the transition to HE. Their words and their frequency, synthesized in a word cloud, illustrate the challenges and demands felt, as well as the mobilization of resources to overcome possible difficulties in their transition and adaptation. In the word cloud, the size of the words increases depending on the number of times they are mentioned. Overall, students used 396 terms to describe the meaning that they give to being an HE student.

**FIGURE 1:**
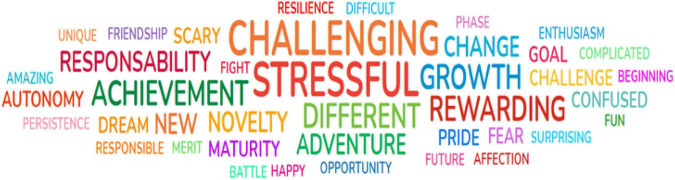
Example of word cloud co-constructed in one of the students’ groups.

A mixed combination of feelings can be identified in a negative (e.g., confused and stressful) or positive sense (e.g., dream and enthusiasm). Considering the words provided by all the students, and the fact that the size of the words increases depending on the number of times they are mentioned, the words that stand out were challenging (*n* = 55), responsibility (*n* = 33), stressful (*n* = 19), change (*n* = 15), and difference (*n* = 14). There are a lot of words and expressions that denote a positive sense, particularly related to new challenges and opportunities, e.g., challenging, responsibility, growth, or adventure. The negative expressions and words are fewer and similar in all five groups, which means they all are experiencing the same difficulties. In this sense, the words and ideas more mentioned were pride, achievement, dream (*n* = 20), autonomy (*n* = 18), devotion and effort (*n* = 14), maturity and growth (*n* = 13), and adventure (*n* = 8). When discussing these results with them, students link these words to the fact that they are newcomers to HE, all is new, and they still need to explore how to manage several aspects related to their academic and personal life. Students also mentioned the uncertainty caused by pandemic (e.g., whether it was safe to go to classes and whether there would be more lockdowns) as an important factor that contributes to their experience of stress and perception of threats. Figure 1 presents the word cloud of one of the five groups to illustrate the activity.

In the second session, “Being a Higher Education student in times of pandemic: academic achievement and success,” students used the *Wooclap* tool to anonymously share their experiences with the group in real-time. Each group built a “Mural of Experiences,” reflecting on “What has been going well” and “What a challenge still is.” Positive experiences and challenges are presented in Table 2. Each student could choose what category to report, both, or none. Their ideas were a starting point to reflect within the group, encouraging students to explore and increase their awareness of academic experiences. The session dynamics promoted sharing personal experiences and strategies to face challenges and identifying good opportunities for supporting and being supported by their peers.

**TABLE 2:** Perceived successes and challenges by the five groups of students.

	No. of times mentioned
What has been going well?	
Adaptation and integration	19
Positive interaction between peers and new friendships	11
Personal interest and motivation for the semester and good results	8
Monitoring the subjects, carrying out group work and preparing for exams/tests	7
Positive interaction between teachers and students	6
Classes work out well in classes, interesting classes	4
Personal organization	2
What is still a challenge?	
Time management and task organization	27
Concentration on online classes	8
Fast pace of learning, keeping the study up to date	6
Tiredness, stress, and anxiety due to group work	5
Meet deadlines	2
Oral presentations at face-to-face classes	4
Adaptation and integration in the class	4
Managing group work and studying for tests	3
Have personal time	1
Total	117

In terms of what was going well, adaptation and integration (*n* = 19) and positive interaction between peers and new friendships (*n* = 11) stand out. In spite of the adverse pandemic situation, students manage to successfully adapt to HE, build positive relationships with both teachers and colleagues, and find relevance in and adjust to academic work. What students find most hard is to organize themselves to successfully complete tasks (*n* = 27) and keep track of the learning pace and the study up to date (*n* = 6). They also find it quite difficult to concentrate on online classes (*n* = 8), which adds to the perceived sense of tiredness, stress, and anxiety (*n* = 5).

Regarding the assessment of dropout risk for this group, Table 3 presents the scores of means and standard deviations in each item and total per scale, namely, Satisfaction with education, Academic exhaustion, and Dropout intentions. The screening instrument was applied at the beginning of the three sessions, but the dropout intentions scale was only applied in the third session as stated before.

**TABLE 3:** Results of the dropout risk screening instrument.

	1st session		2nd session		3rd session	
	*M*	*SD*	*M*	*SD*	*M*	*SD*
Satisfaction with education						
I am satisfied attending this university.	4.55	0.64	4.53	0.62	4.51	0.77
I am satisfied with the education I receive from this university.	4.10	0.77	4.27	0.61	4.15	0.78
My family is pleased with the education I am receiving at this university.	4.34	0.77	4.40	0.67	4.30	0.78
I would recommend this university to a close friend.	4.61	0.61	4.47	0.60	4.54	0.77
Total (4 items)	17.60	2.17	17.67	2.11	17.50	2.81
Academic exhaustion						
I am emotionally exhausted studying this course.	2.71	1.03	2.70	1.08	2.46	1.05
Studying or attending class is stressful for me.	2.68	1.12	2.62	1.11	2.64	1.04
I feel exhausted due to course activities.	2.50	.83	2.53	.87	2.35	1.02
I feel stressed every morning about going to university for another day.	1.94	1.04	2.05	1.11	1.93	1.00
Total (4 items)	9.82	3.19	9.90	3.36	9.38	3.35
Dropout intentions						
I am thinking of changing course.	-		-		1.60	1.17
I have already spoken with friends and/or family about leaving higher education.	-		-		1.36	0.89
I feel insecure / indecisive about continuing to study.	-		-		1.41	0.89
I am thinking in the possibility of dropping out of higher education.	-		-		1.29	0.77
Total (4 items)	-		-		5.66	2.84

Considering 2.5 as the intermediate level of a 5-point Likert-type format scale, most students report feeling satisfied with the educational experience at the university. It is also worthwhile noticing stability of values per item and in total during the first semester (in the three sessions). The levels of emotional exhaustion due to academic activities are less than or close to 2.5. In the third session, it seems to occur a drop in the exhaustion levels. As expected, dropout intentions on the first semester present low levels, above 2.0 in the 5-point Likert-type scale. Considering the profile of these students in previous academic years, as many are not in a first-option grade course, the higher mean on the item “I am thinking of changing course” is predictable.

The students’ evaluation of the sessions, carried out at the end of each session, was very positive. Students were asked to position themselves regarding the content covered, the activities performed, and the duration of the session, using a five-point Likert-type scale of 1 (not suitable) to 5 (very suitable). Table 4 presents descriptive results concerning these three aspects of the sessions.

**TABLE 4:** Students’ evaluation of the three sessions.

	1st session		2nd session		3rd session	
	*M*	*DP*	*M*	*DP*	*M*	*DP*
Contents	4.70	0.56	4.59	0.53	4.61	0.54
Activities	4.63	0.69	4.50	0.54	4.54	0.52
Duration	4.45	0.69	4.36	0.72	4.34	0.68

The overall evaluation of the sessions, considering the content covered, the activities carried out, and the duration of the sessions, was quite positive, with the average student evaluation score always above 4. The first session received a more positive evaluation, which may reflect some initial enthusiasm or higher expectations with interpersonal meetings in pandemic times. When asked about their interest in participating in similar sessions in the following academic year, most students express interest (81%).

## Discussion

With the increasing expansion and democratization of HE, more students from traditionally excluded sociocultural and socioeconomic groups arrive in HE. However, democratizing access does not equate democratizing academic success, as these students present several difficulties when they have to face HE challenges (Adabaş and Kaygin, 2016; Casanova et al., 2018; Tight, 2019). This study illustrates more problems in academic adaptation and higher academic failure and dropout rates in low sociocultural subgroups (Díaz-Mujica et al., 2019; Casanova et al., 2021b). The problem is accentuated when these students are also not in a program and/or in an institution of first vocational option, due to a *numerus clausus* system on access (Fonseca et al., 2014; Ferrão and Almeida, 2018). This situation is more frequent in human and social sciences, including Education programs. This can be explained by fewer opportunities for employment, but also by students often coming from less favored sociocultural backgrounds.

Our study corroborates the evidence that difficulties increase with the negative impact of COVID-19 on students’ academic experiences, achievement, and wellbeing (Hasan and Bao, 2020; Schleicher, 2020; Wang and Zhao, 2020; Farnell et al., 2021). Loneliness, fear, and depressive experiences have increased during the pandemic crisis (Wang and Zhao, 2020; Charles et al., 2021; Schiff et al., 2021). Students from less privileged families, namely, students in Education programs (ObservatoriUM, 2017, 2018), present more difficulties, which justifies paying more attention to their adjustment process to HE. A pilot version of a program to support academic adaptation of first-year students was implemented in the academic year of 2020/2021, and successive changes and improvements have been introduced for a final version. Three sessions were implemented in the first semester, creating opportunities for exploring and sharing cognitions, emotions, and experiences among students, to prevent feelings of maladjustment and psychological discomfort often found in the first weeks at the university.

Results of the program suggest that in the initial weeks, students describe their academic experiences in terms of challenges and demands felt, presenting negative and positive sentences to explore those experiences, which is in accord with previous research (Pascarella and Terenzini, 2005; Kuh et al., 2008). In addition, notwithstanding the difficulties, most students are excited and enter HE with enthusiasm, positive expectations, and a desire to be successful in learning activities and social interactions (Kuh et al., 2008; Diniz et al., 2018; Casanova et al., 2021a; Wangrow et al., 2022). These positive feelings and expectations explain the majority of optimistic words and sentences students use in the first session of the program, oriented to explore the initial academic experiences related to the transition to university. The homogeneity in the discourse that expresses negative feelings may be an indicator that most students present the same difficulties when facing the demands and challenges of being in HE. This strong similarity in students’ feelings and thoughts may be related to common academic and sociocultural backgrounds. The majority of students on Education under graduation programs come from families with low socio-educational-economic backgrounds, are seldom in courses of first vocational option, and present a low GPA (classifications from secondary schools and access exams).

Some weeks later, a large number of students feel well, have positive interactions with colleagues and teachers, and perceive good learning strategies and high achievement. However, some students present difficulties in task organization, procrastination, presentation of work in face-to-face classes, or problems of concentration on online classes, in addition to tiredness, stress, and anxiety due to academic activities and group work. These difficulties are quite frequent in research concerning students’ adaptation to university (Häfner et al., 2015; Lindblom-Ylänne et al., 2017). It is not uncommon for students to perceive significant differences in teaching in secondary school and at the university. Without textbooks for curricular units and without parents’ supervision, students must develop more autonomy and self-regulation on learning and on daily activities.

Satisfaction with the university and the education program is higher and more stable during the first semester. Inversely, the levels of emotional exhaustion are lower, and in our study, there is a decrease when we compared the initial weeks and the end of the semester. This decrease may be interpreted as progressive students’ adjustment to academic challenges. Combining the high levels of satisfaction with low levels of emotional exhaustion at the end of semester strongly reduces dropout intentions. This result may be an impact of program sessions where students can express their academic difficulties and share alternatives to solve them, provided they register a high level of satisfaction with the contents, activities, and duration of the three sessions of the program implemented. For example, the construction of the word cloud facilitated access to students’ perspectives in real-time, promoting the debate about personal and academic expectations, tolerance, and peer support. Sharing their thoughts in a group, under the safety of anonymity, allows the normalizing of feelings and thoughts.

Some limitations can be presented. First, the program is still in its initial or pilot phase of construction, and it is expected that in the next 2 years, it will take on a more definitive version of activities, contents, and intervention strategies, always focusing on the difficulties of adaptation and academic needs of first-year students. The information collected and the initial evaluation of the three sessions highlight the need for increasing the number of sessions. The intervention can be extended to the second semester, e.g., taking into consideration the students’ academic achievement at the end of the first semester. Another limitation is the non-inclusion in this pilot phase of a more controlled assessment design, eventually comparing groups of students who do or do not attend the sessions. Finally, this intervention program will have to be more thoroughly grounded in intervention models and procedures, namely, clarifying its objectives and choosing the activities and strategies that prove to be more effective.

## Conclusion

Students’ transition and adaptation to HE are the phenomena that have been increasingly studied to reduce the attrition and the risk of failure, dropout, or mental health problems. These concerns were suddenly worsened with the pandemic restrictions. Several lockdowns and the return to campus were lived with strong feelings of fear, uncertainty, and loss of sense of normality in students’ daily lives. In this sense, HE institutions were required to implement actions to support students in this period, recognizing the necessity of quickly assessing and responding to students’ needs.

This article presents a support program for students enrolled in two Education courses at a public university in northern Portugal during the first semester of 2020/2021. It was implemented with the aim of promoting academic success and preventing dropout in the first year. Three sessions were developed, focusing on demands, challenges, and difficulties of the transition, difficulties of the academic adaptation and academic performance, and monitoring the dropout risk.

The main results point to student satisfaction with the content, the activities, and duration of the sessions. Students report not only high satisfaction levels with HE attendance, but also some emotional exhaustion due to the academic activities. In view of these results, some measures can be advanced. For example, considering the low socio-cultural background of these students, it seems important to offer them the opportunity to attend activities of a cultural nature, in articulation with teaching, to expand their experiences of cultural growth that will also enhance their wellbeing. Another important measure is ensuring that these students have access to the required technological and didactical resources and develop digital competencies. The pandemic heightened the socioeconomic difficulties of already struggling families seeking to keep their offspring in HE. An important role for the Pedagogical Council and for the presidency of the Institute of Education has been to support students in this area, namely, by lending technological equipment. In addition, given the fact that some students present difficulties in task organization and time management, it would be important to offer training in soft skills, such as learning to manage time and learning to study, in tandem with training in digital skills. Finally, the course coordination should promote the articulation of the learning and assessment tasks that are proposed to the students so that there is no work overload. The continuity of the program is currently being promoted, with some improvements in its planning to ensure a more definitive version of the program in coming years.

## Data Availability Statement

The original contributions presented in the study are included in the article/supplementary material, further inquiries can be directed to the corresponding author/s.

## Ethics Statement

The studies involving human participants were reviewed and approved by University of Minho Ethics Committee (Ref. BI042754054). The participants provided their written informed consent to participate in this study.

## Author Contributions

JC, AG, MM, and LA participated in study conceptualizing, data analysis, and article writing, editing, and revision. JC implemented the program sessions and data collection. All authors contributed to the article and approved the submitted version.

## Conflict of Interest

The authors declare that the research was conducted in the absence of any commercial or financial relationships that could be construed as a potential conflict of interest.

## Publisher’s Note

All claims expressed in this article are solely those of the authors and do not necessarily represent those of their affiliated organizations, or those of the publisher, the editors and the reviewers. Any product that may be evaluated in this article, or claim that may be made by its manufacturer, is not guaranteed or endorsed by the publisher.

## Footnotes

1
www.wooclap.com
